# Role of Najran University Scholarship Students in the United Kingdom in cultural bridging and civilizational dialog

**DOI:** 10.1371/journal.pone.0350745

**Published:** 2026-06-12

**Authors:** Talib bin Ahmed Al-Hamami

**Affiliations:** Full Professor of Da’wah and Islamic Culture, Faculty of Shari’ah and Fundamentals of Religion & Sharia, Educational and Humanitarian Research Center, Najran University, Najran, Saudi Arabia; King Abdulaziz University Faculty of Medicine, SAUDI ARABIA

## Abstract

This study explores the role of Najran University scholarship students in the United Kingdom in promoting cultural bridging and civilizational dialog. To achieve this objective, a structured questionnaire was designed and administered. The instrument demonstrated high validity and reliability, with a Cronbach’s alpha coefficient of 0.879. The study sample consisted of 59 Saudi scholarship students from Najran University currently studying in the United Kingdom. The questionnaire was distributed during the second semester of the 2024–2025 academic year. Taking into consideration the reported limitations of the study, the analysis revealed that Najran University scholarship students in the United Kingdom actively contribute to cultural bridging and civilizational dialog within academic institutions in their host country. Their engagement extends beyond the university setting, encompassing residential life and community interactions, where they help promote Saudi cultural and civilizational values. Furthermore, they play a vital role in fostering cross-cultural relationships with international peers. Statistical analysis revealed no significant differences and very weak effect sizes in terms of gender, age, marital status, academic level, or field of study. Additionally, the study identified key mechanisms and strategies utilized by Najran University scholarship students to introduce and represent Saudi culture and to foster constructive civilizational engagement within the multicultural context of the United Kingdom.

## Introduction

International student mobility is widely recognized as a major site of intercultural exchange, where students both adapt to host society norms and contribute elements of their home cultures to university communities. The experience of international students represents a process of mutual cultural learning: students engage in a new cultural environment, learn its social norms and behavioral values, and at the same time present their own cultures and values to the local university community [1]. Through everyday academic and social interaction, these exchanges can support intercultural understanding and shape cultural perceptions within global higher education environments [2–4].

Among the modern tools of cultural transmission and civilizational interaction is the international scholarship program, which plays a pivotal role in both developing human capital and promoting values in scholarship hosting countries. In this context, Saudi male and female students abroad are seen not only as academic participants but also as cultural representatives who embody and convey Saudi Arabia’s values, beliefs, and traditions. Accordingly, Najran University scholarship students in the United Kingdom are situated at the intersection of academic participation and cultural representation, where their interactions may contribute to intercultural understanding with host communities and international peers [3,5,6].

Contemporary studies emphasize that students from Arab and Islamic backgrounds often assume an informal ambassadorial role, contributing to intercultural dialog through conduct, communication, and participation in multicultural academic spaces [7]. At the same time, scholarship students’ effectiveness in cultural representation is shaped by cross cultural adaptation and communication competence, which develop through sustained engagement in academic and social settings [8,9]. Intergroup contact research further indicates that meaningful interaction across groups is associated with improved attitudes and reduced stereotyping, supporting the role of campus encounters in strengthening mutual understanding [10]. Recent research further confirms that international students can foster trust based relationships between societies through everyday academic and social engagement in host countries [3,5].

Despite this growing body of work on international students’ adaptation and intercultural contact, there remains limited research that examines Saudi scholarship students specifically as active cultural mediators—focusing not only on adjustment challenges, but on the concrete means and strategies they use to bridge cultures and promote civilizational dialog in host environments. UK focused research highlights adaptation dynamics and identity negotiation [4], but fewer studies analyze Saudi students’ proactive cultural bridging practices as forms of intercultural dialog and ethical representation [5,6].

To address this gap, this study examines how Najran University scholarship students in the United Kingdom enact cultural bridging and civilizational dialog with host communities and international students, and which mechanisms and strategies they perceive as most impactful in representing Saudi culture and Islamic ethical values.

This study adopted Kim’s Integrative Theory of Cross Cultural Adaptation, which posits communication as the central mechanism through which individuals adapt and develop cultural competence [8,9]. Intergroup contact theory is used to interpret how sustained interaction may expand intercultural networks and reduce stereotypes [10]. UNESCO’s framing of intercultural dialog informs the study’s emphasis on respect, acceptance, and mutual understanding as foundations for constructive exchange [11].

For conceptual clarity, culture is defined as the set of values, principles, morals, religious rulings, and behaviors that a scholarship student acquires from parents, faith, teachers, mentors, and community. In contrast, civilization refers to the material manifestations of this culture, including the application of scientific and technological advancements to improve human living conditions. Civilizational dialog is operationally defined as the exchange of views with others from different cultures, along with a commitment to respect, acceptance of others, and the promotion of mutual understanding and social peace [12]. Saudi scholarship students represent cultural mediation. Operationally, cultural mediation is defined as the ability to interpret differences among diverse cultures, reduce misunderstandings, and enhance mutual understanding in order to live according to shared civilizational values among peoples [13].

In this study, cultural bridging is examined across key contexts of students’ lived experience (academic interaction, social engagement in residential/community settings, and relationships with international peers), aligning with adaptation and contact perspectives while emphasizing ethical dialog and representation [9–11]. This approach also resonates with scholarship highlighting internationalization as both an opportunity for exchange and a space where cultural identities are negotiated through daily participation in academic life [2,14].

Accordingly, this study seeks to identify the means of cultural bridging and civilizational dialog employed by Najran University scholarship students to introduce Islam’s values, ethics, and civilizational principles as represented through Saudi culture, and to determine the strategies used to promote constructive intercultural understanding in host contexts. To that end, the study has the following objectives:

(1) to elucidate the role of Najran University scholarship students in fostering cultural bridging and civilizational dialog in the United Kingdom;
(2) to promote Saudi cultural and civilizational values abroad;
(3) to identify the most impactful strategies and tools used in cultural bridging and civilizational dialog; and
(4) to reveal significant differences in the perceptions of Najran University scholarship students the basis of academic specialization, study level, gender, marital status, and age.

This research seeks to answer the following questions:

1. What is the role of Najran University scholarship students in cultural bridging and civilizational dialog within the academic environment of host countries?
2. How do Najran University scholarship students contribute to enhancing civilizational dialog within residential and social interaction contexts in host communities?
3. To what extent do Najran University scholarship students influence the promotion of cultural understanding and civilizational dialog with international students outside the Kingdom?
4. What mechanisms and strategies do Najran University scholarship students employ to transmit Saudi cultural values within host communities?
5. Are there statistically significant differences in the perceptions of Najran University scholarship students regarding their role in cultural bridging and civilizational dialog on the basis of academic specialization, study level, gender, marital status, and age?

## Review of the literature

A review of related literature reveals that while several studies have investigated aspects of the scholarship experience, few have examined Saudi students’ roles in promoting their national culture and civilizational values abroad. Al-Qa’id [15] identified four phases in the scholarship experience, including a period of cultural fascination. Al-Toum [16] reported that most students maintain strong cultural and religious identities abroad, with older and more advanced students reporting lower levels of cultural insecurity. Al-Dosari and Mukhaymir [17] highlighted the cultural and behavioral challenges of intercultural engagement, whereas Al-Asmari [18] emphasized the importance of language proficiency for effective integration. However, these studies focus primarily on personal, academic, or psychological dimensions of the study‑abroad experience and do not provide a comprehensive analysis of how Najran University scholarship students function as agents of cultural communication. These studies, although valuable, did not offer a comprehensive analysis of how Saudi students serve as agents of cultural communication. This study seeks to fill that gap by providing an in-depth analysis of the strategies and tools used by Saudi students to represent and transmit Saudi cultural identity in host societies.

More recently, Saudi- and UK-focused research has begun to explore intercultural challenges and communication practices among Saudi students overseas. For example, Bin Obaid [6] examined the experiences of Saudi English language learners in the United Kingdom, emphasizing the role of cultural awareness, intercultural communication skills, and confidence‑building interventions in supporting students’ successful engagement with host communities. While this study sheds light on communication challenges, it does not explicitly address Saudi students’ broader civilizational or cultural ambassadorial roles.

International scholarship research more broadly provides important insights into the cultural experiences of students abroad. For example, Newsome and Cooper [4] documented the cultural and social adjustment patterns of international students in the United Kingdom, highlighting challenges such as culture shock, limited interaction with local students, and the importance of adaptive strategies for establishing meaningful cross‑cultural relationships. These findings are relevant to Saudi students in the UK, who face similar adaptation processes while simultaneously carrying the responsibility of representing their national culture.

More recent studies of Arab students at UK universities confirm that students from Arab and Islamic backgrounds continue to negotiate identity, belonging, and representation while engaging in intercultural dialog within academic and social settings [5].

Similarly, An and Chiang [1] examined international students’ cultural adaptation in China, identifying key dimensions such as cultural empathy, open‑mindedness, emotional stability, social flexibility, and language proficiency. Their framework helps explain how international students develop the intercultural competencies necessary for cultural bridging, offering a useful theoretical lens for assessing how Saudi students engage with host communities.

Recent empirical research reinforces the relevance of these competencies, demonstrating that intercultural skills, social participation, and communication practices are central to students’ ability to act as cultural mediators in host societies [14].

At a broader level, the internationalization of higher education, as discussed by Lumby and Foskett [2], positions students as active participants in cultural exchange and negotiation. Their work highlights how cultural identity, power dynamics, and institutional environments shape students’ engagement in intercultural communication, reinforcing the importance of understanding Najran University scholarship students not only as learners but also as cultural contributors within global academic ecosystems. Recent reviews of internationalization practices further emphasize the need to move beyond mobility statistics and adaptation challenges toward examining students’ ethical, cultural, and civilizational contributions to host communities [3].

The reviewed literature predominantly addressed specific dimensions of the scholarship experience but lacked a comprehensive exploration of Saudi students’ roles in promoting their national culture and civilizational dialog. The integration of recent international and UK‑based studies demonstrates that although intercultural interaction and adaptation are well‑documented phenomena, Saudi students’ intentional role in cultural representation, value transmission, and civilizational dialog remains underexplored.

This study stands out by offering an in‑depth analysis of the strategies, practices, and communicative tools employed by Saudi students to represent and convey Saudi cultural identity in their host societies. This study seeks to fill the existing research gap by examining how these students actively participate in cultural bridging and civilizational dialog through their behavior, dialog, social engagement, and embodiment of Saudi and Islamic values.

## Methodology

### The study adopted a descriptive‒analytical approach

#### Study population.

**Fig 1:**
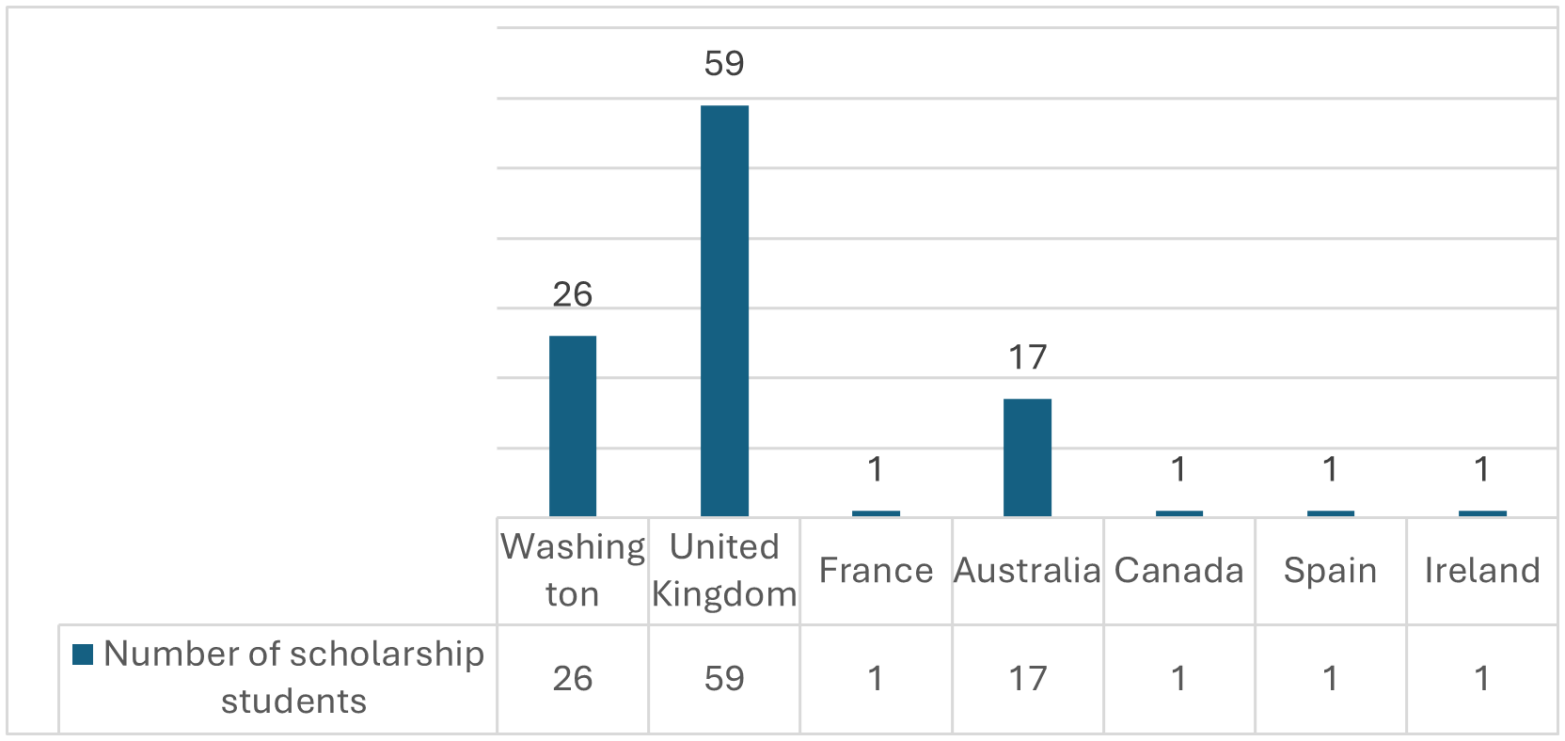
Distribution of the study population by scholarship destination.

The research targeted Najran University scholarship students enrolled in master’s and doctoral programs, including both those who have returned from and those currently residing in the United Kingdom. The sample covered active students during the period from 2020–2025. Data collection began immediately after written ethical approval and a written consent form were obtained from the respondents. The questionnaire was administered online. The participants were contacted through both official and informal communication channels to maximize reach among eligible Najran University scholarship students in the United Kingdom. The official channels included university email communication, whereas the informal channels included social networking platforms and mobile communication (e.g., messaging/phone contact). The recruitment period lasted for two weeks; an extended end date had initially been planned as a contingency in case response rates were insufficient, but it was not required because the targeted responses were obtained within the actual recruitment window (08/06/2025–22/06/2025). Participation was voluntary, and the questionnaire was self-administered; for transparency and to prevent duplicate submissions, participants were permitted to complete the questionnaire only once by restricting access through their university email (one response per email). Email addresses were used solely to enforce the single-submission rule and were not retained or linked to the response dataset, and responses were used exclusively for research purposes. Fig 1 shows the distribution of the study population.

#### Sample of the study.

**Fig 2:**
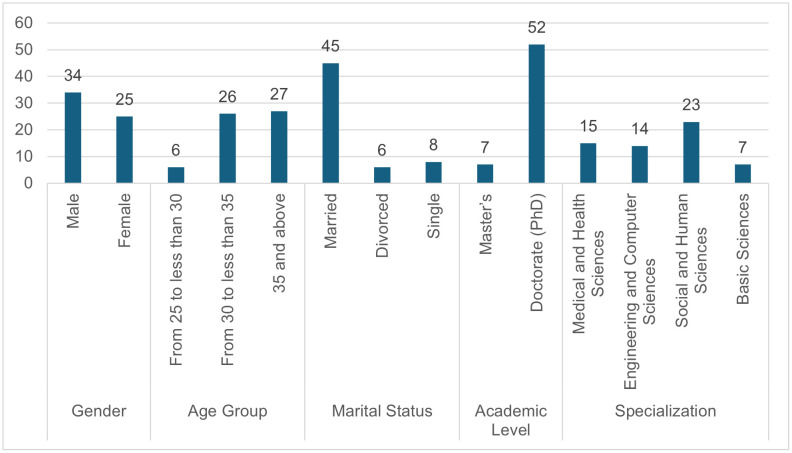
Distribution of the study sample according to demographic variables.

The study employed a purposive sampling approach, targeting Najran University scholarship students enrolled in postgraduate programs (masters’ and PhD) during the 2020–2025 scholarship period. Data were collected exclusively from students studying in the United Kingdom, as it hosts the largest proportion of Najran University scholarship students, as indicated in Fig 2. The questionnaire was administered during the second semester of the 1446 AH/2025 academic year.

The focus on the 2020–2025 period reflects the increased demand for overseas scholarships relative to internal scholarship options during these years. This rise is attributed to the university’s encouraging and incentive‑based initiatives, as well as students’ increased awareness of international university opportunities through the annual Global Universities Exhibition held in Riyadh.

#### Ethical considerations.

Ethical approval for this study was obtained in writing from the Permanent Committee for Research Ethics at Najran University (Approval No. 4600078798, dated 7/6/2025). Data collection was conducted during the second semester of the 1446 AH/2025 academic year, from 08/06/2025–22/06/2025. Prior to participation, the respondents were provided with informed consent information, and participation proceeded only after written consent was obtained. Participation was voluntary, and the respondents were informed that they could decline to participate without any consequence.

To protect participants’ privacy and confidentiality, the questionnaire was self-administered online, and no identifying personal information was collected for analytical purposes. To enhance transparency and prevent duplicate submissions, access was restricted to one response per university email account; however, email addresses were used solely to enforce the single-submission rule and were not retained or linked to the response dataset. All the data were used exclusively for research purposes.

In addition, for transparency, this research reveals the use of generative AI as a support tool for tasks such as proofreading and editing, rephrasing and organizing ideas, and drafting/formatting sections (e.g., results and references), as well as assisting with the initial analysis of qualitative content and searching for relevant academic sources.

#### Instrument of the study.

To achieve the study’s objectives, answer the study’s questions, and collect data, the questionnaire was prepared in its initial form by reviewing previous studies related to the topic of the current study. The questionnaire in its initial form was developed by reviewing previous studies addressing the topic of the current research. These included the study by Al-Tamimi [19], which examined the problems faced by scholarship students pursuing postgraduate studies abroad from Prince Sattam bin Abdulaziz University in Al-Kharj; the questionnaire in that study consisted of six dimensions. It also included the study by Al-Toum [16], which investigated the attitudes of Saudi students studying abroad toward the impact of scholarship programs on their cultural security; its questionnaire comprised 54 items covering all aspects of the study. The current questionnaire consisted of three parts. The first part addressed the scholarship students’ personal data. The second part included the roles to be measured, consisting of (25) statements formulated according to the five‑point Likert scale and distributed across three domains: (1) the role of scholarship students in bridging the cultural gap within the academic environment, (2) promoting cultural values in housing contexts and community interaction, and (3) building cultural relationships with international students. The third part included five questions about the mechanisms and strategies used by Najran University scholarship students in the United Kingdom to introduce Saudi culture and values to the communities of the host country (the United Kingdom). For each item, response options were presented in a multiple‑choice format.

#### Face validity.

The questionnaire was presented to five experts and specialized reviewers: a professor of creed and da’wah at Cairo University; an associate professor of education at Najran University; an associate professor of foundations of education at Najran University; an associate professor of da’wah at King Fahd University of Petroleum and Minerals; and an associate professor of measurement and evaluation at Najran University. This was done to ensure the soundness of item wording and the extent to which each statement belongs to the domain under which it falls. On the basis of the reviewers’ feedback, some statements were reworded when comments were received regarding their phrasing.

In addition, five statements were excluded because several reviewers agreed that they were unclear, with an agreement rate of 80% according to the agreement formula. In the first dimension, there were originally ten items, which were revised and reduced to seven items. The following item was excluded: “I am keen to learn about other cultures and convey them to my colleagues in my home country.” In the second dimension, there were nine items, which were merged into seven items, including: “I strive to break down cultural barriers between my culture and that of the host country.” The third dimension originally consisted of eight items, which were also merged into seven items, including: “I participate in activities, discussions, or dialogs with my neighbors in the host country to introduce them to Saudi values.” Accordingly, the questionnaire achieved face validity.

#### Internal consistency of the questionnaire.

After the questionnaire was administered to the pilot sample (20 male and female scholarship students), the internal consistency was calculated by computing the correlation coefficients between each section of the questionnaire and the total score of the questionnaire. Table 1 presents these coefficients.

**Table 1:** Correlation coefficients between questionnaire scores and total scores.

Section	Correlation Coefficient	Significance Level
First Section	0.931	0.01
Second Section	0.888	0.01
Third Section	0.876	0.01

It is evident from Table 1 that all correlation coefficients are statistically significant at the 0.01 level, indicating that the questionnaire has strong internal consistency.

As shown in Table 2, all reliability coefficients fall within acceptable ranges, indicating that the questionnaire has satisfactory internal consistency. Accordingly, the instrument is considered valid and appropriate for use in the present study. See the final version of the questionnaire in S1 Appendix.

**Table 2:** Distribution of internal consistency coefficients (item–domain and item–total correlations) for the questionnaire items and the domains as a whole.

No.	Item	First domain: Bridging the cultural gap within the academic environment	Second domain: Promoting cultural values in residential/community contexts	Third domain: Building cultural relationships with international students	Total
1	I am aware of Saudi cultural values.	0.545**	—	—	0.367**
2	I actively participate in academic discussions to clarify Saudi values and culture.	0.770**	—	—	0.649**
3	I contribute to organizing academic events to introduce my peers to Saudi culture.	0.737**	—	—	0.739**
4	I strive to correct misconceptions about Saudi culture within the academic environment.	0.410**	—	—	0.324*
5	I try to overcome cultural barriers through academic collaboration and projects.	0.695**	—	—	0.628**
6	I regularly engage in cultural discussions with my international peers from the host country.	0.547**	—	—	0.524**
7	I attempt to integrate Saudi cultural elements into my academic projects.	0.417**	—	—	0.339**
8	I try to incorporate Saudi cultural elements into student activities.	0.686**	—	—	0.542**
9	I am keen to participate in community activities to introduce others to Saudi values.	—	0.779**	—	0.698**
10	I work on strengthening relationships with my neighbors and roommates.	—	0.666**	—	0.545**
11	I contribute to enhancing mutual understanding and bridging cultural gaps between my culture and that of the host country.	—	0.753**	—	0.651**
12	I strive to leave a positive impression of the Saudi community through my daily interactions.	—	0.369**	—	0.312*
13	I respect different values and traditions without compromising my cultural identity.	—	0.289*	—	0.269*
14	I participate in activities, discussions, or dialogs with my neighbors in the host country.	—	0.653**	—	0.554**
15	I utilize social communication channels to present a positive image of Saudi Arabia.	—	0.511**	—	0.528**
16	I offer advice to new Saudi students on how to adapt to the new environment while preserving their cultural identity.	—	0.652**	—	0.655**
17	I make an effort to learn about the cultures of international students and share them with my fellow Saudi colleagues.	—	—	0.595**	0.460**
18	I actively contribute to correcting and changing misconceptions about Saudi Arabia among international students.	—	—	0.387**	0.463**
19	I participate in discussions and dialogs with international students to explain the values and principles of my culture.	—	—	0.640**	0.600**
20	I regularly participate in various cultural activities to introduce international students to my culture.	—	—	0.818**	0.673**
21	I strive to build positive relationships with international students from diverse cultural backgrounds.	—	—	0.658**	0.592**
22	I have participated in volunteer or community projects aimed at bridging cultural gaps.	—	—	0.775**	0.642**

Note: “—” indicates that the item does not belong to that domain (not applicable).

Significance: * p < 0.05, ** p < 0.01.

#### Questionnaire reliability.

The reliability of the questionnaire was calculated using Cronbach’s alpha, where the alpha value across all dimensions of the questionnaire reached (0.879). This coefficient indicates that the reliability is statistically acceptable. At the subscale level, Domain 1 showed acceptable internal consistency (α = 0.748), whereas Domains 2 and 3 yielded borderline coefficients (α = 0.686 and α = 0.673), which should be interpreted cautiously. Accordingly, the questionnaire in its final form consisted of three parts. The first part included general information about the scholarship students. The second part addressed the roles to be measured, comprising (22) items distributed across three dimensions: (the role of scholarship students in bridging the cultural gap within the academic environment, promoting cultural values in housing contexts and community interaction, and building cultural relationships with international students). Each item was accompanied by response options based on a five-point Likert scale (strongly agree, agree, neutral, disagree, and strongly disagree). The third part included three multiple-choice questions about the mechanisms and strategies used by Najran University scholarship students in the United Kingdom to introduce Saudi culture and values to the societies of their host countries. Table 3 presents the values of the reliability coefficients.

**Table 3:** Reliability coefficients for each questionnaire section.

No.	Section	Reliability Coefficient
1	The role of scholarship students in bridging cultural gaps within the academic environment	0.748
2	Promoting cultural values in housing and social interaction contexts	0.686
3	Building cultural relationships with international students	0.673
Overall questionnaire		0.879

Accordingly, the questionnaire, in its final form, consisted of three parts. The first part included general information about the scholarship students. The second part included the roles to be measured, comprising (22) statements distributed across three domains: (1) the role of scholarship students in bridging the cultural gap within the academic environment, (2) promoting cultural values in residential contexts and community interactions, and (3) building cultural relationships with international students. For each statement, response options were provided according to a five‑point Likert scale (Strongly Agree, Agree, Neutral, Disagree, and Strongly Disagree). The third part included three multiple‑choice questions about the mechanisms and strategies used by Najran University scholarship students in the United Kingdom to introduce Saudi culture and values to the host society. The level of need was determined on the basis of Table 4.

**Table 4:** Degree of agreement according to the lower and upper limits of the means of scholars’ responses.

Degree of Agreement	Lower Limit	Upper Limit
Strongly Disagree	1	Less than 1.8
Disagree	1.8	Less than 2.6
Neutral	2.6	Less than 3.4
Agree	3.4	Less than 4.2
Strongly Agree	4.2	5

#### Data analysis.

To analyze the study data and answer the research questions, a combination of descriptive and inferential statistical methods was employed. First, frequencies and percentages were used to describe the distribution of the study sample across the demographic variables (e.g., specialization, academic level, gender, marital status, and age). Next, means and standard deviations were calculated for each item, each domain, and the overall questionnaire to determine the general level of agreement and the variability in participants’ responses. To ensure the quality of the instrument, Cronbach’s alpha was computed to assess the reliability (internal consistency) of each domain and of the questionnaire as a whole, while Pearson correlation coefficients were used to verify internal consistency validity by examining the associations between domain scores and the total questionnaire score.

Before selecting inferential tests, the distribution of scores was examined within each comparison group using the Shapiro–Wilk test, supported by inspection of distributional characteristics. The Shapiro–Wilk test was selected because it is widely regarded as more powerful and informative than the Kolmogorov–Smirnov test for detecting deviations from normality in small samples, while acknowledging that all formal normality tests have limited sensitivity when subgroup sizes are very small [20,21]. Accordingly, decisions regarding the use of parametric or nonparametric procedures were based on a combination of statistical testing and substantive judgment regarding distributional shape, rather than on significance testing alone.

For comparisons involving two independent groups, namely gender and academic level, the independent samples t‑test was applied when the normality assumption was reasonably satisfied and when variance homogeneity was acceptable, as assessed using Levene’s test. The use of parametric testing was considered acceptable in these cases because t‑tests and ANOVA procedures are generally robust to moderate departures from normality when applied to continuous composite scores, particularly when the objective is to compare group means rather than estimate population parameters [20,22]. Nevertheless, given the presence of very small and uneven subgroup sizes (e.g., Master’s students), the results of these analyses were interpreted with caution.

For comparisons involving three or more independent groups, a one‑way ANOVA was used to examine differences according to academic specialization and age when distributional assumptions were judged to be acceptable. However, for marital status, because normality was not achieved in at least one subgroup, the Kruskal–Wallis test was employed as the appropriate nonparametric alternative. This conservative approach was adopted to reduce the risk of assumption violations, particularly in the presence of small subgroup sizes, where parametric estimates may be unstable [20].

Moreover, Eta-squared (η²) was used to calculate the effect size for the following variables: (gender, major, rank, and age). Epsilon-squared (ε²) $\varepsilon^{\text{2}}=\frac{H-k+\text{1}}{n-k}$ was used to calculate the effect size for the marital status variable. Eta-squared (η²) classification: (0.01–0.06)=Small (weak), (> 0.06 to < 0.14)=Medium, (≥ 0.14)=Large; Epsilon-squared (ε²) classification: ES < 0.01 – Very small, 0.01 <= ES < 0.06 – Small, 0.06 <= ES < 0.14 – Medium, ES >= 0.14 – Large.

It is also important to acknowledge that some subgroup sizes in the study were very small (e.g., n = 6–7), which substantially limits statistical power and reduces the likelihood of detecting true group differences even when they exist. Consequently, non‑significant findings should not be interpreted as definitive evidence of equivalence between groups, but rather as an absence of statistically detectable differences under conditions of limited sensitivity [23,24]. For this reason, conclusions drawn from subgroup comparisons were framed cautiously, and null findings were interpreted in light of these methodological constraints.

### Study results

#### Role of Najran University Scholarship Students in Cultural Bridging and Civilizational Dialog within the Academic Environment of Host Countries.

**Fig 3:**
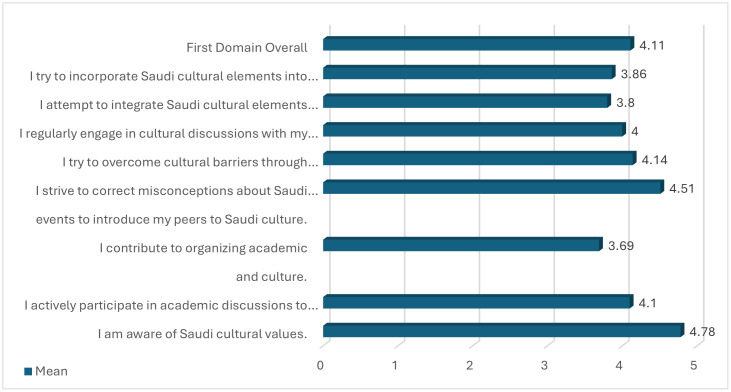
The role of scholarships in bridging cultural gaps within the academic environment.

To answer this question, the mean responses of the sample members were calculated for each statement within the first domain and for the domain as a whole. Fig 3 presents these means.

An analysis of the results presented in Fig 3 indicates that the mean responses of the scholarship students across the statements of the first domain range between 3.69 and 4.78, reflecting generally positive perceptions. Although not all mean scores exceeded 4.0, most items demonstrated moderate to high levels of agreement. The highest mean score was recorded for the statement “I am aware of Saudi cultural values” (M = 4.78), followed by “I strive to correct misconceptions about Saudi culture” (M = 4.51), indicating strong agreement on these aspects. In contrast, lower mean scores were observed for items related to organizing academic events (M = 3.69) and integrating Saudi cultural elements into activities (M = 3.80–3.86), suggesting relatively weaker agreement compared to other items. The overall mean score for the first domain was 4.11, which reflects a general tendency toward agreement among participants regarding the statements within this domain, demonstrating the sample’s agreement with the statements in this domain as a whole. These findings indicate that Najran University scholarship students at Najran University in the United Kingdom effectively fulfill their role in cultural bridging and civilizational dialog within the academic environment of their host countries, as reflected in the items of the first domain of the questionnaire designed for this purpose.

#### Najran University Scholarship Students’ Contribution to Enhancing Cultural Bridging and Civilizational Dialog within the Contexts of Housing and Community Interaction in Host Societies.

**Fig 4:**
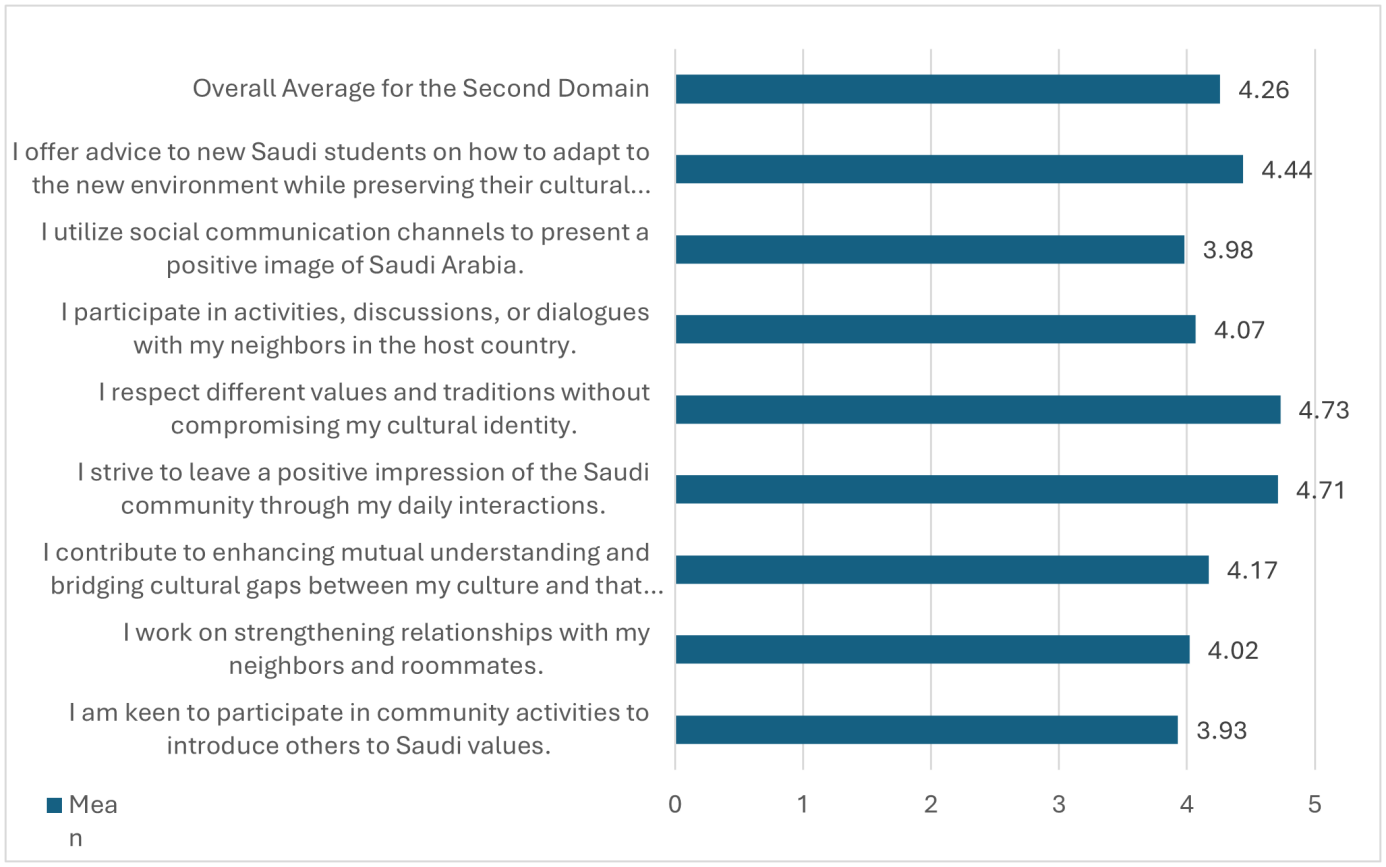
Promoting cultural values in housing and social interaction contexts.

To answer this question, the mean responses of the sample members were calculated for each statement within the second domain, as well as for the overall domain. Fig 4 presents these average values.

The findings presented in Fig 4 indicate that the mean scores of respondents’ answers within the second domain ranged from 3.93 to 4.73, reflecting generally positive attitudes among the participants. Strong agreement was evident for the statements “I respect different values and traditions without compromising my cultural identity” (M = 4.73) and “I strive to leave a positive impression of the Saudi community through my daily interactions” (M = 4.71). Additionally, the item related to offering advice to new Saudi students recorded a relatively high mean score (M = 4.44), indicating a strong sense of supportive engagement. Other items showed moderate agreement, with mean scores ranging between 3.93 and 4.17, suggesting positive but comparatively lower endorsement. The overall mean score for the second domain was 4.26, reflecting a general tendency toward agreement among participants regarding the statements in this domain. These results indicate that Najran University scholarship students in the United Kingdom are effectively fulfilling their role in enhancing cultural bridging and civilizational dialog within residential and community interaction contexts, as outlined in the statements of the second domain of the questionnaire.

#### Najran University Scholarship Students’ Contribution to Enhancing Cultural Understanding and Civilizational Dialog with International Students outside the Kingdom of Saudi Arabia.

**Fig 5:**
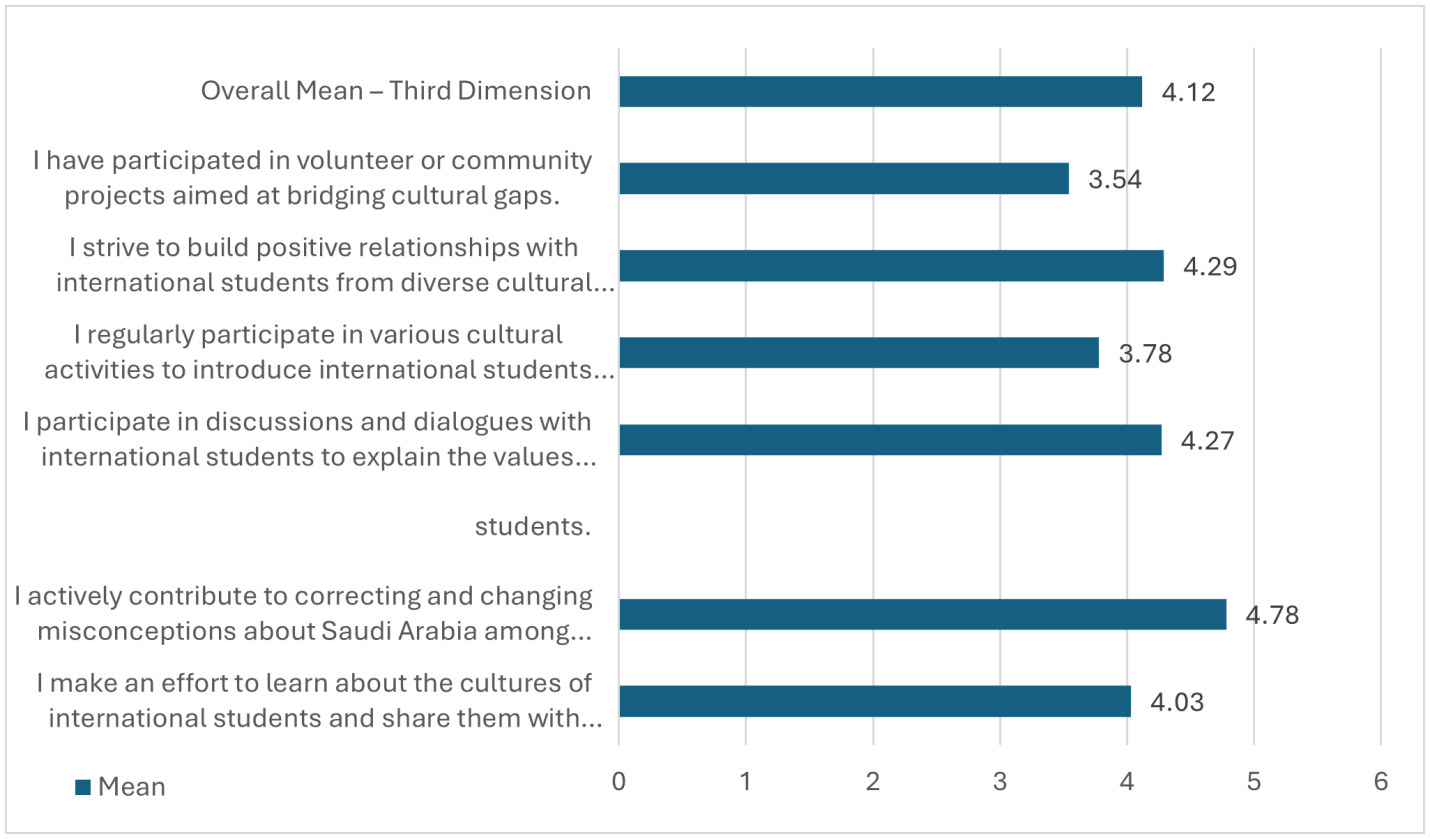
Building cultural relationships with international students.

To answer this question, the means of the participants’ responses were calculated for each item in the third domain, as well as for the domain as a whole. The results are presented in Fig 5.

The results presented in Fig 5 show that the mean responses of the scholarship students for the items comprising the third dimension ranged from 3.54 to 4.78, indicating generally positive perceptions, though the level of agreement varied across items. “I actively contribute to correcting and changing misconceptions about Saudi Arabia among international students” (M = 4.78), reflecting strong agreement with this statement. Two additional items—building positive relationships with international students (M = 4.29) and participating in discussions and dialogs to explain Saudi values (M = 4.27)—demonstrated high but slightly lower levels of agreement. In contrast, comparatively lower mean scores were observed for participation in volunteer or community projects (M = 3.54) and regular participation in cultural activities (M = 3.78), suggesting moderate agreement on these activities.

The overall mean score for the third dimension was 4.12, which reflects a general tendency toward agreement among participants with the statements in this dimension. These findings indicate that Najran University scholarship students in the United Kingdom play an active role in promoting cultural understanding and civilizational exchange, particularly through dialog, relationship‑building, and correcting misconceptions, although participation in organized community and volunteer initiatives appears comparatively less emphasized. Furthermore, the overall mean score for the entire questionnaire was 4.16, indicating a generally positive level of agreement with the survey content as a whole.

#### Mechanisms and Strategies used by Najran University Scholarship Students to Promote Saudi Culture and Values within Host Countries.

To address this question, the frequencies of participants’ responses to three specific questions related to the mechanisms and strategies employed by Najran University scholarship students to transmit Saudi culture and values in their host countries were calculated. The following section presents the frequency distribution for each of the three items.

**Fig 6:**
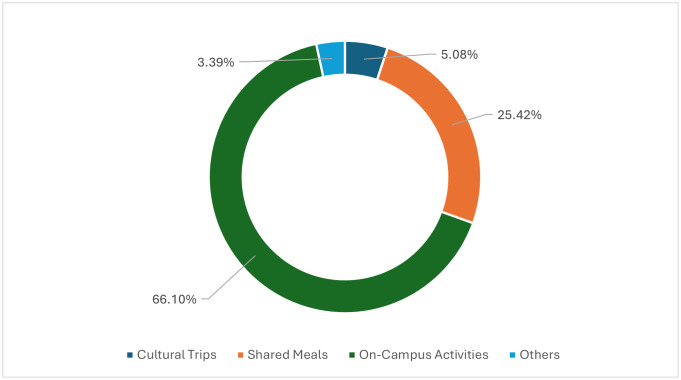
Activities through which Najran University scholarship students contribute to promoting cultural understanding with international students in the host country.

Fig 6 clearly shows that the activity through which Najran University scholarship students contribute the most to enhancing cultural understanding and intercultural communication with international students in the host country is on-campus activities, followed by shared meals.

**Fig 7:**
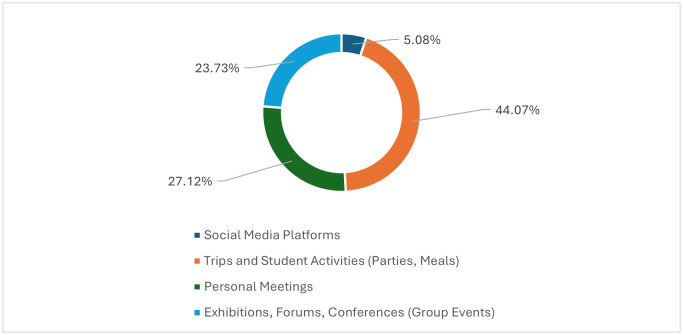
Methods used by Najran University scholarship students to introduce Saudi culture to others in the host country.

Fig 7 clearly shows that the method most commonly used by Najran University scholarship students to introduce Saudi culture to others in the host country is student activities, including parties, trips, and meals, followed by personal meetings.

**Fig 8:**
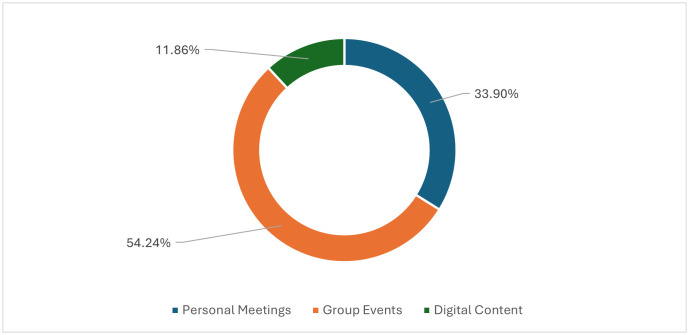
The most influential method, according to Najran University scholarship students, for introducing Saudi culture in the host country.

Fig 8 clearly shows that the most effective method for introducing Saudi culture in the host country, according to Najran University scholarship students in the United Kingdom, is group events, followed by personal meetings and then digital content.

#### Differences in the Responses of Najran University Scholarship Students Regarding their Role in Cultural Bridging and Civilizational Dialog.

To answer this question, the mean responses of the sample individuals were calculated for each of the aforementioned variables. Table 5 presents these means.

**Table 5:** Means and standard deviations of sample individuals’ responses according to the research variables.

Variable	Variable Levels	Count	Mean	Standard Deviation
Academic Specialization	Medical and Health Sciences	15	4.14	0.308
	Engineering and Computer Sciences	14	4.16	0.31
	Social and Humanities Sciences	23	4.18	0.49
	Basic Sciences	7	4.19	0.52
Academic Level	Master’s	7	4.01	0.33
	Doctorate	52	4.19	0.42
Gender	Male	34	4.09	0.32
	Female	25	4.25	0.49
Marital Status	Married	45	4.20	0.37
	Divorced	6	4.09	0.189
	Single	8	4.02	0.68
Age	Age 25 to less than 30	6	4.23	0.49
	Age 30 to less than 35	26	4.17	0.45
	Age 35 and above	27	4.15	0.36

From Table 5, it is evident that there are apparent differences between the mean responses of the sample individuals attributed to the research variables (academic specialization, academic level, gender, marital status, and age).

### Specialization variable

As shown in Table 6, all the Shapiro–Wilk p values for the specialization groups are greater than 0.05, indicating that the total scores are approximately normally distributed across all the specialization categories; thus, the use of parametric tests (e.g., one-way ANOVA) is appropriate.

**Table 6:** Test of normality (specialization variable).

	Specialization	Shapiro‒Wilk		
		Statistic	df	Sig.
Total	Medical and Health Sciences	.971	15	.875
	Engineering and Computer Sciences	.953	14	.603
	Social and Humanities Sciences	.965	23	.576
	Basic Sciences	.828	7	.077

*. This is a lower bound of true significance.

a. Lilliefors Significance Correction

Table 7 shows that the mean total scores across specializations are very close (ranging from 4.14–4.19) and confirms that these small differences are not statistically significant (F(3,55) = 0.036, p = 0  .991); therefore, academic specialization does not affect students’ responses regarding their role in cultural bridging and civilizational dialog.

**Table 7:** Significance of differences in sample responses attributed to academic specialization.

	N	Mean	Std. Deviation	Std. Error	95% Confidence Interval for Mean		Minimum	Maximum
					Lower Bound	Upper Bound		
Medical and Health Sciences	15	4.1394	.30755	.07941	3.9691	4.3097	3.64	4.77
Engineering and Computer Sciences	14	4.1591	.31021	.08291	3.9800	4.3382	3.64	4.82
Social and Humanities Sciences	23	4.1759	.49931	.10411	3.9600	4.3918	2.86	5.00
Basic Sciences	7	4.1948	.52270	.19756	3.7114	4.6782	3.50	4.68
Total	59	4.1649	.40934	.05329	4.0582	4.2715	2.86	5.00
	Sum of Squares		df		Mean Square		F	Sig.
Between Groups	.019		3		.006		.036	0.991
Within Groups	9.699		55		.176			
Total	9.719		58					

In addition, the effect size for the academic specialization was reported using Eta-squared as shown in Table 8. The results showed that the η² of  .002 indicates that academic major explains only about 0.2% of the variance in the total score, which represents a weak (small/negligible) effect size. In practical terms, differences in the total score across majors are minimal.

**Table 8:** Measures of association (major).

	Eta	Eta Squared
Total * major	.045	.002

### Academic level

To determine the significance of the apparent differences in the sample responses attributed to the academic level variable, tests of normality were applied to determine a suitable test for differences. Table 9 presents the results of this test.

**Table 9:** Tests of normality (academic level).

	Academic level	Shapiro‒Wilk		
		Statistic	df	Sig.
Total	Master’s	0.986	7	0.983
	Doctorate	0.973	52	0.292

*. This is a lower bound of the true significance.

a. Lilliefors Significance Correction.

All Shapiro–Wilk p values for both academic levels (Master’s and Doctorate) are greater than 0.05, indicating that the total scores are approximately normally distributed across the two academic groups, which supports the use of parametric testing (independent-samples t‑test) to compare their means.

The independent-samples t‑test in Table 10 shows no statistically significant difference in total mean scores between Master’s (M = 4.01) and Doctorate students (M = 4.19), t(57) = −1.047, p = .300, and because Levene’s test is non‑significant (p = .507), the equal-variances result is appropriate (the 95% CI for the mean difference also includes zero).

**Table 10:** Independent samples test (academic level).

	Academic level	N		Mean		Std. Deviation			Std. Error Mean			
Total	1.00	7		4.0130		33166			.12535			
	2.00	52		4.1853		.41717			05785			
		Levene’s Test for Equality of Variances		t test for Equality of Means								
		F	Sig.	t	df		Sig. (2-tailed)	Mean Difference		Std. Error Difference	95% Confidence Interval of the Difference	
											Lower	Upper
Total	Equal variances assumed	.446	.507	-1.047-	57		.300	-.17233-		.16467	-.50207-	.15741
	Equal variances not assumed			-1.248-	8.781		.244	-.17233-		.13806	-.48583-	.14118

In addition, the effect size for the academic level was reported using Eta-squared as shown in Table 11. The η² of 0.019 indicates that academic level explains approximately 1.9% of the variance in the total score. According to the η² classification you provided (0.01–0.06 = weak/small), this represents a weak (small) effect size. In practical terms, differences in total scores across academic levels are present but limited in magnitude.

**Table 11:** Measures of association (academic level).

	Eta	Eta Squared
Total * academic level	.137	.019

#### Gender.

To determine the significance of the apparent differences in the sample responses attributed to the gender variable, tests of normality were applied to determine a suitable test for differences. Table 12 presents the results of this test.

**Table 12:** Tests of normality (gender).

	Gender	Shapiro‒Wilk		
		Statistic	df	Sig.
Total	1.00	.971	34	.483
	2.00	.929	25	.085

*. This is a lower bound of the true significance.

a. Lilliefors Significance Correction.

All Shapiro–Wilk p‑values for both gender groups are greater than 0.05 (male: p = .200/.483; female: p = .082/.085), indicating that the total scores are approximately normally distributed for males and females, which supports the use of a parametric independent‑samples t‑test to compare gender means.

The independent-samples t‑test in Table 13 indicates that although females reported a higher mean score (M = 4.26) than males did (M = 4.10), this difference is not statistically significant (t(57) = −1.518, p = .134), and because Levene’s test is non‑significant (p = .058), the equal‑variances assumption result is appropriate.

**Table 13:** Independent samples t-test (gender).

		Gender		N	Mean			Std. Deviation			Std. Error Mean		
Total		1.00		34	4.0963			.31843			.05461		
		2.00		25	4.2582			.49978			.09996		
		Levene’s Test for Equality of Variances		t-test for Equality of Means									
		F	Sig.	t		df	Sig. (2-tailed)		Mean Difference	Std. Error Difference		95% Confidence Interval of the Difference	
												Lower	Upper
Total	Equal variances assumed	3.732	.058	-1.518-		57	.134		-.16193-	.10665		-.37549-	.05164
	Equal variances not assumed			-1.422-		38.003	.163		-.16193-	.11390		-.39251-	.06866

In addition, the effect size for gender was reported using Eta-squared as shown in Table 14. The η² of  .039 means that gender explains about 3.9% of the variance in the total score. Based on your η² classification (0.01–0.06 = weak/small), this represents a weak (small) effect size. Although there is some association between gender and the total score, the magnitude is small, indicating that gender has a limited practical influence on participants’ total scores in this study.

**Table 14:** Measures of association (gender).

	Eta	Eta Squared
Total * gender	.197	.039

### Marital Status

To determine the significance of the apparent differences in the sample responses attributed to the marital status variable, tests of normality were applied to determine a suitable test for differences. Table 15 presents the results of this test.

**Table 15:** Tests of normality (marital status).

	Social	Shapiro‒Wilk		
		Statistic	df	Sig.
Total	Married	0.980	45	0.632
	Divorced	0.728	6	0.012
	Single	0.922	8	0.448

*. This is a lower bound of the true significance.

a. Lilliefors Significance Correction.

Table 15 shows that the married and single groups have non‑significant normality tests (p > .05), but the divorced group shows a significant departure from normality (Shapiro–Wilk p = .012), so the normality assumption is not met overall, and a non‑parametric test (Kruskal–Wallis) is therefore appropriate for comparing marital‑status groups.

From Table 16, it is evident that there are no statistically significant differences in the responses of the sample individuals attributed to marital status.

**Table 16:** Kruskal‒Wallis test (marital status).

Marital Status Level	Count	Mean Rank	Chi- Square	Degrees of Freedom	Significance Level
Married	45	31.44	0.623	2	0.732
Divorced	6	24.17			
Single	8	26.25			

Using the Kruskal–Wallis statistic (H = 0.623), number of groups (k = 3), and total sample size (N = 59), the estimated effect size using epsilon-squared (ε²) is ε² ≈ 0.00, indicating a negligible/very weak effect.

#### Age.

To determine the significance of the apparent differences in the sample responses attributed to the age variable, tests of normality were applied to determine a suitable test for differences. Table 17 presents the results of this test.

**Table 17:** Tests of normality (age).

	Age	Shapiro‒Wilk		
		Statistic	df	Sig.
Total	25 to less than 30 years old	0.945	6	0.704
	30 to less than 35 years old	0.962	26	0.429
	35 years old and above	0.963	27	0.432

*. This is a lower bound of true significance.

a. Lilliefors Significance Correction.

All the Shapiro–Wilk p‑values for the three age groups are greater than 0.05, indicating that the total scores are approximately normally distributed across age categories, which supports the use of a parametric one‑way ANOVA to test for age‑based differences.

According to Table 18, the descriptive results show that the mean total scores across the three age groups are very similar (25– < 30: M = 4.23, 30– < 35: M = 4.17, 35 + : M = 4.15), and the one‑way ANOVA confirms that these differences are not statistically significant (F(2,56) = 0.085, p = .918), indicating that age does not significantly influence respondents’ perceptions of their role in cultural bridging and civilizational communication.

**Table 18:** Significance of differences in sample responses attributed to age.

	N	Mean	Std. Deviation	Std. Error	95% Confidence Interval for Mean		Minimum	Maximum
					Lower Bound	Upper Bound		
25 to less than 30 years old	6	4.2273	0.49710	0.20294	3.7056	4.7489	3.50	4.77
30 to less than 35 years old	26	4.1661	0.44978	0.08821	3.9844	4.3478	2.86	5.00
35 years old and above	27	4.1498	0.36140	0.06955	4.0069	4.2928	3.45	4.82
Total	59	4.1649	0.40934	0.05329	4.0582	4.2715	2.86	5.00
	Sum of Squares	df	Mean Square	F	Sig.			
Between Groups	0.030	2	0.015	0.085	0.918			
Within Groups	9.689	56	0.173					
Total	9.719	58						

In addition, the effect size for gender was reported using Eta-squared as shown in Table 19. The η² of  .003 means that age explains approximately 0.3% of the variance in the total score. Based on the adopted η² classification (0.01–0.06 = weak/small), this value represents a very weak (negligible) effect size. Although there is a measurable association between age and the total score, its magnitude is minimal, indicating that age has virtually no practical influence on participants’ total scores in this study.

**Table 19:** Measures of association (age).

	Eta	Eta Squared
Total * age	.055	.003

## Discussion

Before interpreting the findings, it is important to emphasize that the conclusions of this study should be viewed as context-specific and exploratory. The study is based on a relatively small sample drawn from a single institution and a single host-country setting, and it relies primarily on self-reported questionnaire data. As a result, the statistical patterns reported here are best interpreted as indicative of students’ perceived practices and tendencies within this particular scholarship context rather than as definitive evidence of broader behavioral outcomes or generalizable national patterns. In addition, self-report measures are susceptible to response tendencies such as social desirability, acquiescence, and ceiling effects—especially when the topics relate to identity, cultural representation, and values—meaning that high mean scores may partly reflect normative expectations rather than only behavioral variance. Importantly, interpretation at the domain level is also moderated by the measurement properties of the subscales: Domains 2 and 3 showed borderline internal consistency (α = 0.686 and α = 0.673), so findings related to residential/community interaction and international-student relationships are treated as more tentative than Domain 1. Accordingly, the findings are interpreted cautiously, focus on the relative patterns across domains and variables rather than absolute score magnitudes, and avoid extending claims beyond comparable populations and settings.

The findings of the present study indicate that Najran University scholarship students in the United Kingdom generally perceive themselves as fulfilling an active and positive role in cultural bridging and civilizational dialog across the three contexts examined in this study: the academic environment, residential life and community interaction, and engagement with international students. The overall mean of the scholars’ responses to the questionnaire was 4.16, indicating a general level of agreement with the questionnaire items. This relatively high overall mean suggests that the respondents view themselves as committed to presenting a positive image of their country and culture while engaging constructively with the host society and other international students.

Consistent with Kim’s Integrative Theory of Cross‑Cultural Adaptation, the high scores imply that students view communication in everyday academic and social interaction as a key mechanism through which they develop intercultural competence and manage adaptation demands [8,9]. Likewise, the strong endorsement of relationship‑building and misconception‑correction aligns with intergroup contact theory, which predicts more positive intergroup perceptions when contact is sustained and meaningful [10]. The particularly elevated responses on cultural understanding/exchange also resonate with UNESCO’s emphasis on intercultural dialog grounded in respect, acceptance, and mutual understanding [11]. However, because these results are self‑reported and potentially influenced by social desirability and common‑method bias, they provide stronger evidence of students’ perceived roles than of objectively verified intercultural impact—thus only partially confirming the theories at the behavioral level.

The high overall score may reflect scholarship students’ strong sense of responsibility toward representing their culture and country, as well as the pride they associate with serving as informal national and cultural ambassadors. It may also be influenced by the preparatory and orientation programs provided by the university prior to departure, which are intended to raise students’ awareness of their role in promoting a positive image of their home country and institution. However, these interpretations should be understood as possible explanations for participants’ responses rather than direct evidence of the actual effectiveness of such roles or programs.

At the same time, these generally high mean scores should be interpreted with caution. The consistently elevated responses across most dimensions—particularly in the third dimension, which focuses on cultural understanding and civilizational exchange—suggest that participants reported a high level of perceived engagement in these roles. Nevertheless, because the findings are based on self-report data from a small sample drawn from a single institution, the results may also reflect social desirability, acquiescence, or identity-related response tendencies. Recent research has shown that self-report surveys measuring prosocial, identity-related, or morally valued behaviors are especially vulnerable to social desirability bias, often producing inflated mean scores and overly positive self-assessments [25,26]. As scholarship students are often aware of their representative role, they may have been inclined to portray themselves as actively engaged in cultural bridging, which may have contributed to elevated self-reported scores [27]. Accordingly, the findings are best interpreted as indicating that the participants perceived themselves as highly engaged in cultural representation and intercultural interaction, rather than as demonstrating that they objectively or consistently fulfilled these roles in practice.

This potential response bias does not invalidate the findings; rather, it suggests that the results should be understood as reflecting perceived roles and self‑assessments rather than solely actual behavioral practices. Methodological research also indicates that reliance on a single self‑report instrument may amplify common method effects, strengthening positive response patterns across dimensions [26,28]. Consequently, while the findings indicate a strong perceived commitment among Najran University scholarship students to cultural representation and intercultural engagement, future research may benefit from incorporating qualitative methods, observational data, or reports from host‑community members to provide a more balanced and comprehensive understanding of students’ cultural bridging behaviors.

More broadly, the results are consistent with previous Saudi and international scholarship research suggesting that students abroad do not function solely as learners adjusting to a new environment, but also as cultural actors who negotiate identity, belonging, and representation in everyday academic and social settings [2,5,16,17]. This interpretation is particularly important in the context of scholarship students, whose experiences often combine cultural adaptation with a perceived responsibility to represent their home country positively.

In the academic setting, the high mean scores suggest that participants perceive the university environment as an important space for cultural communication and representation. Their responses indicate active involvement in clarifying Saudi cultural values, correcting misconceptions, and using academic dialog and collaboration as channels for intercultural exchange. This finding is consistent with Bin Obaid [6], who emphasized the importance of intercultural communication skills, confidence, and cultural awareness in enabling Saudi students in the United Kingdom to engage successfully with others. It also aligns with Lumby and Foskett’s [2] argument that internationalization in higher education places students within broader processes of cultural exchange and negotiation, where identity and institutional context shape communication practices.

Moreover, the present findings can also be interpreted in light of An and Chiang’s [1] framework of intercultural adaptation, which highlights cultural empathy, open-mindedness, social flexibility, and language competence as central to successful engagement in host environments. These competencies appear to be reflected in the participants’ reported efforts to explain their culture and maintain constructive academic relationships.

In residential and community contexts, respondents also reported strong agreement regarding their efforts to leave a positive impression through daily behavior, respect different values and traditions without compromising their own identity, and contribute to mutual understanding with neighbors and members of the host community. However, because this evidence comes from Domain 2, which showed borderline internal consistency (α = 0.686), these findings should be interpreted cautiously as perceived tendencies rather than precise estimates of a tightly unified construct. These results support the findings of Newsome and Cooper [4], who showed that the cultural and social experiences of international students in the United Kingdom are shaped significantly by everyday interaction, social adjustment, and the ability to build meaningful cross-cultural relationships beyond formal academic spaces.

The current results also correspond with Abdulazeez et al. [5], who found that Arab international students in UK universities continue to negotiate belonging, identity, and cultural representation in both academic and social settings. In this sense, the students’ role in cultural bridging appears to extend beyond formal representation into daily interpersonal conduct, which becomes an important medium through which culture is communicated and understood.

In addition, the findings of the second dimension suggest that scholarship students view respectful coexistence, advice-giving, social participation, and positive communication as key features of their cultural role in the host society. This supports the broader argument that intercultural engagement is often enacted not only through formal events, but also through routine acts of interaction, cooperation, and relationship-building. In this regard, Hofhuis et al. [14] reinforce the importance of communication practices, social participation, and intercultural interaction in facilitating adaptation and mediation between cultures.

The third domain provides further evidence that Najran University scholarship students in the United Kingdom perceive themselves as active contributors to civilizational dialog with international peers. High responses on items related to dialog, relationship-building, correcting misconceptions about Saudi Arabia, and sharing values with international students indicate that participants see intercultural exchange as a reciprocal process rather than a one-sided process of adaptation. Nevertheless, because Domain 3 reliability was marginal (α = 0.673), conclusions drawn from this domain are presented conservatively and interpreted as indicative perceptions that would benefit from further measurement refinement and triangulation in future research. This interpretation is strongly supported by Mittelmeier et al. [3], who argue that meaningful internationalization should be understood not merely in terms of student mobility or adaptation challenges, but also in terms of students’ ethical, cultural, and civilizational contributions to host communities.

Accordingly, the present findings suggest that Najran University scholarship students do not simply adapt to the host context; rather, they also attempt to shape intercultural understanding by acting as mediators between cultures and by challenging stereotypes through dialog and social engagement.

Overall, the discussion of the findings in relation to previous literature indicates that the role of scholarship students in cultural bridging is multidimensional. It involves academic participation, interpersonal conduct, social integration, and intercultural dialog, all of which contribute to representing national culture and fostering mutual understanding. At the same time, the high self-reported scores require careful interpretation in light of possible social desirability bias. Therefore, the study contributes to the literature by showing that Saudi scholarship students in the United Kingdom perceive themselves not only as recipients of an international educational experience, but also as active participants in cultural exchange and civilizational dialog.

With respect to demographic differences, the statistical analyses revealed no statistically significant differences and weak effect sizes in Najran University scholarship students’ perceptions attributable to gender, academic specialization, academic level, age, or marital status, suggesting that cultural bridging behaviors are shaped more by shared scholarship experiences, common institutional environments, and individual engagement patterns than by these background variables. This pattern is consistent with research showing that gender and field/major often do not significantly predict intercultural sensitivity/competence when students share comparable learning environments, while interactional exposure variables (e.g., international friendships, contact experiences) are more explanatory [29–31]. Similarly, evidence indicates that international student adjustment outcomes (e.g., acculturation/sociocultural adaptation) may not vary by marital status in some higher education contexts, reinforcing the interpretation that demographic background does not necessarily differentiate intercultural experiences under common institutional conditions [32].

The absence of statistically significant differences in the responses of the study sample may be explained by the fact that scholarship students are exposed to comparable academic demands and multicultural interaction opportunities in the United Kingdom, share a similar sense of cultural representation responsibility, and respond to broadly framed questionnaire items that capture general cultural‑bridging behaviors rather than gender‑specific patterns. Empirical work supports the idea that direct cultural exposure and engagement—such as intercultural contact, training, language proficiency, and international friendship networks—tends to explain more variance in intercultural competence/sensitivity than demographic variables like gender [29,31,33]. In addition, broader syntheses of international student research show that gender differences in adjustment indicators (e.g., acculturative stress) are often inconsistent across studies and may be non‑significant overall, lending support to the present finding of demographic similarity within a shared host‑country context [34].

In summary, the results strengthen the argument that Najran University scholarship students in the United Kingdom contribute meaningfully to cultural bridging and civilizational dialog through everyday academic participation, community engagement, and peer interactions. This interpretation is aligned with evidence that intercultural development is frequently associated with everyday interactional opportunities and contact quality rather than fixed demographic characteristics [31,33]. Moreover, the lack of demographic differences (across most variables) supports the interpretation of this role as a shared and consistent practice among scholarship students within this specific setting, consistent with studies in which demographic effects (e.g., gender/age) are absent while contextual or engagement variables account for observed variation [29,35].

## Conclusion

The results highlighted that Najran University scholarship students in the United Kingdom fulfill the role assigned to them in cultural bridging and civilizational communication within the academic environment, as well as in residential contexts and community interactions. The study also concluded that there are no statistically significant differences and very weak effect sizes attributable to the variables of specialization, gender, academic level, age, and marital status, which may be explained by the similarity of the academic environment and the relative homogeneity of experiences and interactions with the host community. Accordingly, this study contributes to identifying the mechanisms and strategies used by Najran University scholarship students in the United Kingdom to introduce and transmit Saudi culture and values to the host society (the United Kingdom). However, conclusions related to residential/community interaction and engagement with international students should be interpreted cautiously because the corresponding subscales (Domains 2 and 3) demonstrated borderline reliability, indicating lower precision at the domain level despite strong overall reliability.

## Limitations of the study

A key limitation affecting the generalizability of the present findings is that the study was confined to Najran University scholarship students in the United Kingdom at the postgraduate level (Master’s and PhD), thereby excluding scholarship students from other Saudi universities and those enrolled in different types of programs across a wider range of international universities. In addition, the sample was restricted to scholarship students located in the UK as the host country rather than other destination countries. This decision was justified by the fact that the UK hosts the largest proportion of Najran University scholarship students.

Moreover, data collection was conducted during the second semester of the 1446 AH/2025 academic year, which may limit the extent to which the findings reflect students’ experiences at other points in time. In addition, the study focused specifically on the scholarship period (2020–2025), given the notable increase in overseas scholarship demand during these years. This increase is attributed to the university’s supportive and incentive-based initiatives, as well as students’ increased exposure to the opportunities and benefits of international universities through the annual Global Universities Exhibition in Riyadh. Consequently, the results should be interpreted within the context of these temporal and institutional boundaries and should not be generalized beyond similar populations and settings without caution.

In addition to the above contextual limits, a measurement-related limitation should be acknowledged. Although the overall reliability of the questionnaire was high (α = 0.879) and the first domain demonstrated acceptable internal consistency (α = 0.748), the second and third domains produced marginal Cronbach’s alpha values (α = 0.686 and α = 0.673). These borderline coefficients may reduce precision at the subscale level, and therefore findings related to Domains 2 and 3 should be interpreted cautiously and conservatively. Furthermore, because Cronbach’s alpha reflects internal consistency rather than construct validity, the instrument is best described as demonstrating adequate overall reliability with some subscale-level limitations rather than being fully “valid” based solely on alpha coefficients.

Furthermore, the consistently high mean scores observed across the questionnaire domains—many exceeding 4.0 and several approaching the upper end of the scale—may reflect ceiling effects and social‑desirability or acquiescence bias, a pattern commonly reported in self‑report studies addressing values, identity, and national or cultural representation. Given that participants share a common institutional affiliation (Najran University) and scholarship status, respondents may have been inclined to present themselves in ways that are consistent with normative expectations, particularly when framing themselves as cultural or national representatives. In addition, the ethical and religious dimensions embedded in concepts such as cultural bridging, respect, and civilizational dialog may cause socially desirable responses rather than purely behavioral variance. To mitigate these effects, the data collection procedures emphasized anonymity and voluntariness: the questionnaire was self‑administered, no identifying information was collected, participation was not linked to academic or supervisory evaluation, and no faculty members or supervisors were present during completion. Nevertheless, the possibility of response bias cannot be fully eliminated and should be considered when interpreting the magnitude of the mean scores.

## Future research

In light of the study’s limitations, the researcher recommends replicating the study with broader and more diverse samples to strengthen the generalizability of the findings. First, because the current research was limited to Najran University scholarship students, future studies should include scholars from multiple Saudi universities to enable institutional comparisons and to determine whether the identified patterns of cultural bridging and civilizational dialog are consistent across different university contexts. Second, since the study focused only on postgraduate students (masters’ and PhD), future research should include undergraduate scholarship students and students enrolled in language institutes or professional training programs, as levels of maturity, exposure, and interaction opportunities may differ across academic stages. Third, as the sample was restricted to the United Kingdom as the host country, future research should expand to other major scholarship destinations—particularly those with large Saudi scholarship populations such as the United States and Australia—to examine whether the host country context (e.g., cultural distance, institutional practices, and community openness) influences students’ cultural representation and civilizational engagement.

Moreover, because data were collected during one academic semester (second semester of 1446 AH/2025), future studies should adopt a longitudinal design that tracks scholarships across different stages of their scholarship journeys (e.g., first arrival, mid‑program, and pre‑return) to capture how cultural bridging behaviors develop over time. In addition, since the present study focused on the scholarship period (2020–2025), future studies may compare cohorts from different periods to examine whether changes in scholarship policies, university incentives, or global conditions affect students’ cultural communication roles.

Furthermore, to address the limitations of using a questionnaire alone, future research should employ mixed methods (e.g., interviews, focus groups, observations, or reflective journals) to gain deeper insight into *how* and *why* students use particular cultural communication strategies and to explore contextual factors that may not emerge through quantitative measures. Finally, to address measurement limitations identified in this study, future research should refine Domains 2 and 3 by reviewing item–total correlations, revising unclear items, and/or adding theoretically relevant items to improve internal consistency, followed by re-estimating reliability.

## Supporting information

S1 Appendix Questionnaire: Role of Najran University Scholarship Students in Cultural Bridging and Enhancing Civilizational Understanding in the United Kingdom. (PDF)
