# Supplementary material for: Role of Najran University Scholarship Students in the United Kingdom in cultural bridging and civilizational dialog
Source: PLoS One. 2026 Jun 12;21(6):e0350745. doi: 10.1371/journal.pone.0350745 (PMC13262832; doi:10.1371/journal.pone.0350745)
Supplement: S1 Appendix — (PDF) [file pone.0350745.s001.pdf]

**Questionnaire: Role of Najran University Scholarship Students in Cultural Bridging and Enhancing Civilizational Understanding in the United Kingdom**

**Dear scholarship student (Brother/Sister) ………………………………,**

Greetings,

This study aims to measure the extent to which Saudi scholarship students contribute to bridging and reducing cultural gaps between their home country (the Kingdom of Saudi Arabia) and the scholarship host country (the United Kingdom), with a focus on the academic environment, housing/residential life, and the community. It also seeks to understand the strategies and mechanisms used by scholars to introduce host communities to Saudi culture and values. We assure you that all the information you provide will be treated with complete confidentiality and will be used for scientific research purposes only. Thank you for your valuable participation in our questionnaire.

**Section One: Personal Information**

**1) Gender**

- ☐ Male
- ☐ Female

**2) Age**

- ☐ Less than 25 years
- ☐ 25–30 years
- ☐ 31–35 years
- ☐ More than 35 years

**3) Marital Status**

- ☐ Single
- ☐ Married

**4) Length of stay in the host country**

- ☐ Less than one year
- ☐ From 1 to 3 years
- ☐ More than 3 years

**5) Academic level**

- ☐ Master's
- ☐ Doctorate (PhD)

**6) Academic specialization**

- ☐ Medical and Health Sciences
- ☐ Engineering Sciences
- ☐ Social and Humanities Sciences
- ☐ Other specializations (please specify): ________________________

**Section Two: Questionnaire Statements (Likert Scale)**

**Response options:**

- **Strongly Agree**
- **Agree**
- **Neutral**
- **Disagree**
- **Strongly Disagree**

**Domain One: The Role of Scholarship Students in Bridging the Cultural Gap within the Academic Environment**

| No. | Item | Strongly agree | Agree | Neutral | Disagree | Strongly disagree |
|---|---|---|---|---|---|---|
| 1. | I am aware of Saudi cultural values. | | | | | |
| 2. | I actively participate in academic discussions to clarify Saudi values and culture. | | | | | |
| 3. | I contribute to organizing academic events to introduce my peers to Saudi culture. | | | | | |
| 4. | I strive to correct misconceptions about Saudi culture within the academic environment. | | | | | |
| 5. | I try to overcome cultural barriers through | | | | | |

| | | | | | | |
|---|---|---|---|---|---|---|
| | academic collaboration and projects. | | | | | |
| 6. | I regularly engage in cultural discussions with my international peers from the host country. | | | | | |
| 7. | I attempt to integrate Saudi cultural elements into my academic projects. | | | | | |
| 8. | I try to incorporate Saudi cultural elements into student activities. | | | | | |

**Domain Two: Promoting Civilizational Values in Residential Contexts and Community Interaction**

| No. | Item | Strongly agree | Agree | Neutral | Disagree | Strongly disagree |
|---|---|---|---|---|---|---|
| 1. | I am keen to participate in community activities to introduce others to Saudi values. | | | | | |
| 2. | I work on strengthening relationships with my neighbors and roommates. | | | | | |
| 3. | I contribute to enhancing mutual understanding and bridging cultural gaps between my culture and that of the host country. | | | | | |
| 4. | I strive to leave a positive impression of the Saudi community through my daily interactions. | | | | | |
| 5. | I respect different values and traditions without compromising my cultural identity. | | | | | |
| 6. | I participate in activities, discussions, or dialogs with my neighbors in the host country. | | | | | |
| 7. | I utilize social communication channels to present a positive image of Saudi Arabia. | | | | | |
| 8. | I offer advice to new Saudi students on how to adapt to the new environment while preserving their cultural identity. | | | | | |

**Domain Three: Building Cultural Relationships with International Students**

| No. | Item | Strongly agree | Agree | Neutral | Disagree | Strongly disagree |
|---|---|---|---|---|---|---|
| 1. | I make an effort to learn about the cultures of international students and share them with my fellow Saudi colleagues. | | | | | |
| 2. | I actively contribute to correcting and changing misconceptions about Saudi Arabia among international students. | | | | | |
| 3. | I participate in discussions and dialogs with international students to explain the values and principles of my culture. | | | | | |
| 4. | I regularly participate in various cultural activities to introduce international students to my culture. | | | | | |
| 5. | I strive to build positive relationships with international students from diverse cultural backgrounds. | | | | | |
| 6. | I have participated in volunteer or community projects aimed at bridging cultural gaps. | | | | | |

**Section Three: Mechanisms and Strategies Used by Najran University Scholarship Students in the UK**

**23) Activities through which you contribute to enhancing cultural understanding with international**

**students in the host country**

- ☐ Cultural trips
- ☐ Shared meals
- ☐ On-campus activities
- ☐ Other: ______________________

**24) Methods used by Najran University scholarship students in the UK to introduce Saudi culture to others in the host country**

- ☐ Social media platforms
- ☐ Student activities (parties, trips, and meals)
- ☐ Personal meetings
- ☐ Exhibitions, forums, conferences (group events)
- ☐ Presentations and documentaries (digital content)

**25) The most influential method, in your view, for introducing Saudi culture in the host country**

- ☐ Personal meetings
- ☐ Group events
- ☐ Digital content
